# Proteomic analysis reveals dynamic changes in cloacal fluid composition during the reproductive season in a sexually promiscuous passerine

**DOI:** 10.1038/s41598-024-62244-3

**Published:** 2024-06-20

**Authors:** Kristýna Míčková, Václav Jelínek, Oldřich Tomášek, Romana Stopková, Pavel Stopka, Tomáš Albrecht

**Affiliations:** 1 Department of Zoology, Faculty of Science, Charles University, Prague, Czech Republic; 2 Institute of Vertebrate Biology, The Czech Academy of Sciences, Brno, Czech Republic; 3 Department of Botany and Zoology, Faculty of Science, Masaryk University, Brno, Czech Republic

**Keywords:** Zoology, Sexual selection, Reproductive biology

## Abstract

Cryptic female choice (CFC) is a component of postcopulatory sexual selection that allows females to influence the fertilization success of sperm from different males. While its precise mechanisms remain unclear, they may involve the influence of the protein composition of the female reproductive fluids on sperm functionality. This study maps the protein composition of the cloacal fluid across different phases of female reproductive cycle in a sexually promiscuous passerine, the barn swallow. Similar to mammals, the protein composition in the female reproductive tract differed between receptive (when females copulate) and nonreceptive phases. With the change in the protein background, the enriched gene ontology terms also shifted. Within the receptive phase, distinctions were observed between proteomes sampled just before and during egg laying. However, three proteins exhibited increased abundance during the entire receptive phase compared to nonreceptive phases. These proteins are candidates in cryptic female choice, as all of them can influence the functionality of sperm or sperm-egg interaction. Our study demonstrates dynamic changes in the cloacal environment throughout the avian breeding cycle, emphasizing the importance of considering these fluctuations in studies of cryptic female choice.

## Introduction

In sexually promiscuous species, postcopulatory sexual selection through sperm competition^1^ and cryptic female choice^2^, constitutes a significant component of egg fertilization. While sperm competition is a widely accepted and relatively frequently studied component of mate choice, cryptic female choice has, until recently, been an unjustly neglected mechanism^3^. However, growing evidence indicates that females are not just passive receivers of sperm as previously thought but can actively choose which sperm to use for fertilization^4^. Cryptic female choice, though a subtle and complex process, has deep evolutionary significance with the potential to impact the entire evolutionary landscape of reproduction. By favoring or disfavoring sperm of specific males, females can influence the traits of their offspring and improve own fitness. However, such selection may also create an evolutionary "arms race" where males develop counter-adaptations to overcome female cryptic choice^3^. Females may actively eject sperm after copulation^5,6^ but most of the selection probably occurs at the morphological, biochemical, and physiological levels in the female reproductive tract, for instance through an interaction between sperm and the fluids secreted by females^7^. Females might also not use the sperm for fertilization immediately after copulation, instead, they can store sperm in specialized structures for extended periods of time^8,9^, which significantly increases the potential for cryptic female choice to operate^8,10^. Females, that could control the paternity of their offspring through cryptic female choice, would be able to favor good or sexy sperm, or most importantly prefer sperm from unrelated and more compatible males to ensure the highest possible fitness of their offspring^3,4^. Therefore, cryptic female choice should not be overlooked, especially in the context of genetic diseases or assisted reproduction.

Despite growing evidence of direct interaction between sperm and the female reproductive tract across species, the exact mechanisms of cryptic female choice remain largely unexplored. The main reason for the lack of understanding the cryptic female choice is that in animals with internal fertilization all processes occur inside the female´s reproductive tract which is inaccessible to direct observation in vivo. Artificial insemination of equal numbers of sperm from different males and subsequent observation of resulting paternity is one way to test cryptic female choice^11^. Another option is to test the effect of fluid from the female reproductive tract on sperm performance as female reproductive fluids are supposed to interact with the ejaculate, mediate sperm migration, ensure sperm survival, and induce their modification^12^. Research on sperm-female fluid interactions has so far mostly focused on externally fertilizing fishes^11,13^ but also on mammals^14,15^ and, more recently, birds^16–19^. There is some evidence that female vaginal or cloacal fluid can impact sperm traits such as motility^17,20,21^ and/or longevity^17,22,23^. Sperm motility and longevity are traits shown to affect sperm competitive abilities and reproductive success of the male^24,25^. More research is needed to fully understand the specific mechanisms underlying cryptic female choice. It is, however, likely that the chemical composition of female reproductive tract secretions plays a role.

The composition of female reproductive fluids is complex and includes inorganic salts, amino acids, proteins, and fatty acids^26,27^, all with potential effect on sperm performance. Compared to seminal fluids^28^, however, the exact composition of fluids from the female reproductive tract remain mostly overlooked even though they represent the environment the sperm must pass through to fertilize the egg. Research has mostly focused on the effect of pH or ionic composition of female reproductive fluids on sperm performance^27,29,30^, but the protein composition of the reproductive tract is also likely to play an essential role. Proteomic analysis provides a comprehensive description of the protein composition of various fluids. There is increasing interest in this topic due to the assumed involvement of proteins in postcopulatory selection—storage, usage, signalization, or fertilization^31^. However, there is still much to learn about the specific proteins involved in sperm x female environment interactions. The composition of female reproductive fluids seems to be highly variable and can differ between females^32,33^ and within individuals, with some changes being related to diseases^34,35^ or pregnancy^36–38^. The occurrence of some proteins or their abundance may also cyclically change during the reproductive cycle^39–41^. This temporal variation in the environment of the female reproductive tract may have a fundamental impact on female fertility and reproductive health. Proteins more abundant in the receptive part of the reproductive tract are often associated with stress or immune responses, but they can also directly affect sperm function and survival or interactions between gametes^40,42^. The specific proteins involved in these changes can differ between species. Research focusing on mammals has provided some insights^39–42^, but information on other groups like birds is still emerging. Although not completely understood, changes in protein abundance may also have a diverse impact on sperm that are exposed to the reproductive fluids after copulation^13^ and can play an important part in female cryptic choice, especially in passerines given their well-documented promiscuous behavior^43,44^.

Bird cloaca is the part of the reproductive tract that initially comes into contact with the male ejaculate, and cloacal fluids correspond to the vaginal environment. The vagina itself is probably the major barrier to sperm, with only 1–2% of sperm being able to adapt to the female environment and pass through the vagina^45^. The proteins and metabolites present in the female reproductive tract during sperm passage and storage probably influence the survival of sperm^46,47^. In birds, some descriptions of uterine fluid^48,49^, oviductal fluid^50^ and cloacal fluid^19^ proteomes are available. However, only the latter study focused on passerines. In this study, we evaluated the protein composition of cloacal fluids sampled from individual female European barn swallows (*Hirundo rustica rustica*) throughout the different phases of their reproductive cycle using label-free proteomics (nLC-MS/MS). Barn swallows are sexually promiscuous songbirds, where approximately 20% of nestlings in up to 40% of nests are sired extra-pair^51,52^. Moreover, the effect of cloacal fluid on sperm motility has been confirmed in this species *in vitro*^53,54^. We assumed that candidate proteins most influencing sperm behavior would be present in the female reproductive tract primarily during the time when they actively mate with males (receptive phase). Thus, our approach, applied in a sexually promiscuous bird species, represents a potentially effective way to identify essential proteins that could influence sperm performance after the copulation, thereby playing a pivotal role in cryptic female choice.

## Methods

### Study area and general field procedure

In 2020 and 2021, adult females of barn swallows were captured at the locality Hamr (49° 3′ 24.217″ N, 14° 46′ 9.361″ E), in South Bohemia, protected landscape area Třeboňsko, to collect cloacal fluid samples in various reproductive phases. Female capture was conducted in the early morning hours prior to the initiation of copulation to ensure the absence of male ejaculate in the samples. Birds were ringed with an aluminium National Museum of Prague ring and with an individual combination of colour rings for easy identification of individuals on the nest that was necessary to determine female reproductive phases. While the exact duration of the receptive phase, during which females copulate with males, in barn swallows remains uncertain, it is generally assumed that in passerines, females are receptive over a period starting ca. 5 days before the laying of the first egg (day 0) until the laying of the penultimate egg in a clutch^55–57^. In this study, we captured females just after their arrival at the locality, a time when they are not yet breeding (hereafter referred to as the prereceptive phase, PRE). These prereceptive samples (n = 12) were taken on average − 10.5 ± 3.3 (SD) days before laying their first egg in a clutch (range − 17 to − 6 days). Subsequent samples from the same females were collected just before or during the egg-laying period, when females copulate regularly (receptive phase—REC). The mean time of sampling was − 0.5 ± 1.7, with a range − 4 to 2 days before/after laying of the first egg in their clutch. The last sampling occurred during incubation or feeding of offspring in the nest (postreceptive phase—POST), with a mean sampling time of 22.3 ± 5.8 (range 12 to 33 days after laying the first egg in a clutch). The reproductive phase of captured females was initially determined based on the development of the brood patch and the appearance of the cloaca^58,59^, and later confirmed precisely by monitoring on the nests at the breeding locality (see also^52,60^). Using the latter approach, it was possible to divide females into two groups—females sampled in the receptive phase before laying their first egg (REC-BL, n = 5) and those already egg-laying (REC-L, n = 7) due to the possible presence of egg-laying proteins that could interfere with the analysis. For the collection of reproductive fluids, we adapted the methodology from^16^. In brief, we obtained cloacal fluids by injecting 8 µl of sterile saline solution (phosphate-buffered saline, PBS) into the cloaca, allowing a 10 s period of mucosal contact, and then withdrawing the solution into a tube. The collected samples were promptly placed and further stored in liquid nitrogen for subsequent proteomic analysis. We declare that all experiments performed for this study were approved by the animal and ethics representatives of The Czech Academy of Sciences and nature conservation authorities (MZP/2020/630/964). All methods were carried out in accordance with relevant regulations and guidelines and reported in accordance with ARRIVE guidelines (https://arriveguidelines.org).

### Sample processing

Female cloacal fluids samples (N = 36 samples from 12 females) were thawed at room temperature after the end of the season and then vortexed. Subsequently, 5 μl of the fluids were mixed with 20 μl of PBS in 0.5 ml tubes and vortexed again. In the next step, a buffer for isolation was added to each tube and samples were left in room temperature for one hour. This procedure was followed by ice-cold acetone (1:4) precipitation, samples centrifugation (14,000 rcf, 10 min at 4 °C) and re-suspension of the dried pellets in the digestion buffer (1% SDC, 100 mM TEAB—pH = 8.5). To determine the protein concentration, the BCA assay kit was used (Fisher Scientific, Waltham, MA, USA). Cysteines in 20 μg of proteins were reduced to a final concentration of 5 mM TCEP (60 °C for 60 min). After trypsin cleavage (i.e., 1/50, trypsin/protein) in 37 °C overnight, peptides were desalted using a Michrom C18 column. The retrieved peptide cations were then transformed into gas-phase ions through electrospray ionization. The analysis was carried out using a Thermo Orbitrap Fusion (Q-OT-qIT; Thermo Fisher, Waltham, MA, USA) under the same condition and setup as previously described^61,62^.

### Bioinformatics

Data were quantified using MaxQuant sotware version 1.6.34. The false discovery rate (FDR) was set to 1% for peptides and proteins identification and minimum peptide length was arranged to the length of seven amino acids. To obtain protein IDs, the Andromeda engine was used for the MS/MS mapping against the UniProt database (zebra finch genome, *Taeniopygia guttata*, UP000007754, 2021). Quantifications were performed with the label-free algorithms^63^ using a combination of multiple unique and razor peptides. Raw data were LFQ normalized and are available as Supplementary Material. Further analysis was conducted in the R software^64^. We remove all proteins which were detected but not quantified (zero abundances), all proteins with less than two unique peptides and all rare proteins from our dataset so only proteins detected in at least three samples of cloacal fluids remained in the dataset. Using the *NormalyzerDE* package^65^, quantile normalization was selected as the most appropriate due to the lowest within-group variance. Sparse Partial Least Squares Discriminant Analysis (sPLS-DA) within the *mixOmics* package^66^ was used to compare the individual phases of reproduction. The sPLS-DA analysis enabled the identification of proteins responsible for the differences between individual phases. The results were evaluated based on the Area Under Curve analysis (AUC). To detect proteins that are responsible for the divergence between fluids from phases, we used the Power Law Global Error Model (PLGEM) for identification of differently expressed proteins and their corresponding *p* value^67^. The results are presented in the form of a volcano plots using *ggplot2* package^68^. Gene ontology (GO) searches for significantly enriched GO terms were performed using Gene Set Enrichment Analysis (GSEA) within the *clusterProfiler* package^69^. GO terms were further searched within the latest chicken database (January 2024)—org.Gg.eg.db. Visualization of the enriched GO terms were conducted using *ggplot2* and related packages in R^68^.

## Results

We collected samples of female cloacal fluids at four reproductive phases (PRE, REC-BL, REC-L, POST) from a total of 12 females of barn swallows. However, two samples had to be excluded from the analysis due to an insufficient number of detected proteins. So, for proteomic analysis we used 12 samples of female cloacal fluids in PRE phase, 11 in REC phases (5 of REC-BL and 6 of REC-L phase) and 11 in POST phase. The raw dataset, extracted from label-free quantification and LFQ normalized, was based on 963 protein identifications. After removing all proteins which were detected but not quantified, all proteins with less than two peptides and all rare proteins, our dataset contained a total of 501 proteins (Supplementary Material). In total, 52 out of 501 proteins were detected in all the samples (e.g., ALB, ANXA1, ANXA2, HSPA5, HSPA8, KRT19, RPS27A).

**Figure 1:**
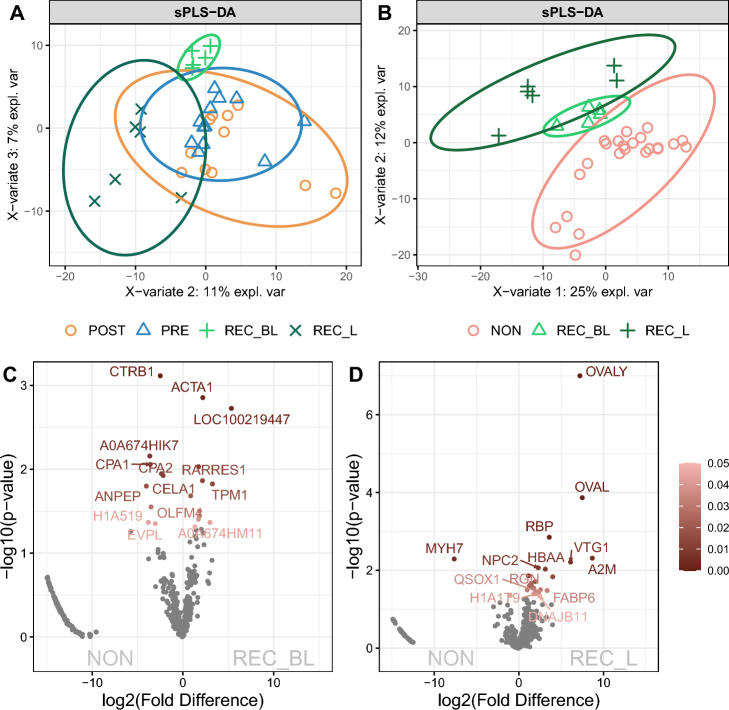
Differences in the proteomic composition of cloacal fluids during the reproductive cycle. Sparse Partial Least Squares Discriminant Analysis (sPLS-DA) shows differences in the proteomic composition of cloacal fluid during reproductive season. Four phases—prereceptive (PRE), receptive before (REC-BL) and during egg laying (REC-L) and postreceptive (POST)—are shown in (**A**). The PRE and POST phases exhibit a high degree of overlap in protein representation; consequently, we merged them into a single phase for subsequent analysis. Three phases (after merging PRE and POST in nonreceptive phase—NON) are illustrated in (**B**). The significantly differentially expressed proteins between NON and REC-BL (**C**) and REC-L (**D**) phases were detected by the Power Law Global Error Model (PLGEM), and they are displayed using the volcano plot. Significance is scaled from light (p < 0.05) to dark (p < 0.00001) brown.

In the first step, we tested whether the protein composition of cloacal fluids is stable or changes during the female reproductive cycle (PRE, REC-BL, REC-L, POST) using the Sparse Partial Least Squares Discriminant Analysis (sPLS-DA). The results showed that there is a variation in the presence of proteins in cloacal fluids within individual phases of reproduction (graphically shown in Fig. 1A, used comp2 and comp3 for their higher informativeness). Using the Area Under Curve analysis (AUC), we discovered that both receptive phases, REC-BL (receptive before laying vs. others—Comp3 (y-axis): AUC = 1, p < 0.001) and REC-L (receptive laying vs others—Comp2 (x-axis): AUC = 1, p < 0.001; Comp3 (y-axis): AUC = 1, p < 0.001), are distinct from others (values for all comparisons are in Supplementary Table S1). The PRE and POST phases are very similar in protein composition, which can be seen from the graphical representation, where the distribution of samples of these two phases overlaps. In the next step, we identified the proteins that are most responsible for the differences between the individual phases of reproduction. The results are displayed as loading values (Supplementary Fig. S1A and S1B) and thus with the relevance of proteins in contribution on each principal component (Comp2 and Comp3) of the sPLS-DA analysis. The same color code as in Fig. 1A was used.

For the following analysis, we decided based on previous results to merge PRE and POST into the nonreceptive phase group (NON) based on their overlap of protein composition. Then, we again compared NON, REC-BL and REC-L using the sPLS-DA analysis. Based on the AUC analysis (graphically shown in Fig. 1B; values for all comparisons are in Supplementary Table S2), the NON phases were the most different from both REC phases—REC-BL and REC-L (nonreceptive vs. others—Comp1 (x-axis): AUC = 0.8, p = 0.002; Comp2 (y-axis): AUC = 1, p < 0.001). At the same time REC phases also differed from others (receptive before laying vs. others—Comp1 (x-axis): AUC = 0.7, p = 0.181; Comp2 (y-axis): AUC = 1, p = 0.039; receptive laying vs others—Comp1 (x-axis): AUC = 0.8, p = 0.009; Comp2 (y-axis): AUC = 1, p < 0.001). The proteins responsible for these differences are shown in Supplementary Fig. S2A and S2B, as well as their relevance in contribution to each principal component (Comp1 and Comp2) of the sPLS-DA analysis.

The proteins that were significantly differentially expressed between phases were detected by the Power Law Global Error Model (PLGEM). Together with the differentially expressed proteins, we obtained their corresponding p values. The results are graphically demonstrated in Fig. 1C,D. In the REC-BL, 13 proteins were more abundant compared to the NON phase (e.g., ACTA1, LOC100219447—myosin heavy chain, A0A674GTS9, RARRES1, TPM1, KRT19) and 17 proteins less abundant (e.g., CTRB1, A0A674HIK7, CPA1-2, CELA1, ANPEP, MYH7). In the REC-L, 23 proteins were more represented (e.g., OVALY, OVAL, RBP, VTG1, A2M, FABP6) and, on the contrary, there was a lower occurrence of MYH7, SPINK5 and TTR compared to the NON phases. Candidate proteins of cryptic female choice were considered to be those that were significantly differently represented (compared to NON) in both REC-BL and REC-L. The occurrence of RBP, RARRES1, and LOC100232544 (serine protease inhibitor 2.1 like) proteins significantly increased in both REC phases. On the contrary, the samples were characterized by a lower occurrence of MYH7.

**Figure 2:**
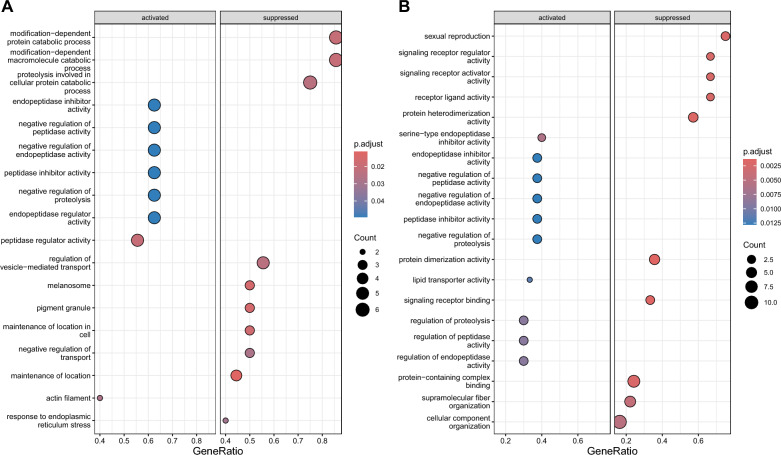
Enriched gene ontology (GO) terms in receptive phase before (**A**) and during (**B**) egg laying from Gene Set Enrichment Analysis (GSEA). The size of the circles indicates the number of genes that have been enriched, while the range of Benjamini–Hochberg corrected p-values is depicted through a gradient from red to blue.

To determine the physiological function of cloacal fluid, we searched the GO terms using Gene Set Enrichment Analysis (GSEA) for the proteins represented in the REC-BL and REC-L phases (graphically shown in Fig. 2A,B). The results allow us to determine which GO terms are activated or suppressed in the given phase. During the REC-L phase expressed proteins are involved in activation of actin filament (GO:0005884) and regulation of proteolysis, e.g. peptidase regulator activity (GO:0061134), negative regulation of proteolysis (GO:0045861) and peptidase activity (GO:0010466), peptidase (GO:0030414) and endopeptidase inhibitor activity (GO:0004866), etc. On the contrary, processes associated with catabolic activity (like proteolysis involved in cellular protein catabolic process (GO:0051603), modification-dependent macromolecule (GO:0043632) and modification-dependent protein (GO:0019941) catabolic process), or regulation of transport, e.g. negative regulation of transport (GO:0051051) and regulation of vesicle-mediated transport (GO:0060627), are suppressed. In the REC-L phase, enriched GO terms, that are activated, are associated with lipid transporter activity (GO:0005319) and also, as in the REC-BL phase, regulation of proteolysis, like regulation of proteolysis (GO:0030162), peptidase (GO:0052547) and endopeptidase activity (GO:0052548), negative regulation of proteolysis (GO:0045861) and peptidase activity (GO:0010466), etc. The suppressed processes then include sexual reproduction (GO:0019953), protein binding (like signaling receptor binding (GO:0005102), protein dimerization (GO:0046983) and heterodimerization (GO:0046982) activity) or signaling (e.g., signaling receptor regulator activity (GO:0030545) and activator activity (GO:0030546) or receptor ligand activity (GO:0048018)).

## Discussion

Our study evaluates changes in the proteomic composition of female reproductive fluids in birds during the reproductive cycle. From all identified proteins, more than a half (284 from 501) can be found in vaginal fluid of mice^70^, including e.g. the most abundant LYZ, LOC100224071 (ovostatin-like), LTF, SPINK5, ALB and ANXA1. Currently, the only one additional study, that has dealt with the composition of cloacal female fluid in passerines, is available^19^, however, the study has a relatively small sample size, and the authors did not account for potential variation in cloacal fluid composition associated with reproductive stages of females. Our study represents a significant advancement in rigorously testing and analyzing the changes of proteomic composition of the fluids from the female reproductive tract during entire reproductive season. Barn swallow females were captured at four distinct phases of reproduction, roughly corresponding to the sperm prereceptive, receptive (before and during egg-laying) and postreceptive phases. This allowed us to assess the within-individual, within-seasonal stability of cloacal fluid proteomic composition and to compare protein profiles during the highly receptive period, when copulations are likely, with periods when females are not fully receptive. Our findings provide clear evidence of shifts in cloacal protein composition of individual barn swallow females. The results indicate that the female reproductive environment is not stable but cyclically changes based on the female's readiness for reproduction. Based on the protein composition of female fluid, we are therefore able to safely distinguish between REC and NON periods. However, even during receptivity, the protein composition of the fluid does not remain unchanged, and in REC-L we can find a number of proteins associated with egg production and egg-laying. Nevertheless, both receptive phases, REC-BL and REC-L create the environment to which male sperm are exposed after copulation. Seasonal changes in the female reproductive environment, associated with changes of female receptivity, have already been demonstrated in other animal species^27,40,41,70^, and notably in humans^39,71^.

During the transition from PRE to REC-BL, the production of some proteins increases, such as ACTA1, LOC100219447 (myosin heavy chain), A0A674GTS9, RARRES1, TPM1, and KRT19. Protein RARRES1 (retinoic acid receptor responder protein 1) is worth mentioning. This protein (sometimes referred to as OCX32) was found in the uterus, where it participates in the mineralization of the eggshell. Further, RARRES1 function as a protease inhibitor is necessary for the egg-sperm interaction during fertilization^50^. TPM1 (tropomyosin 1) is involved in morphogenesis and is a component of smooth muscle cells^72^. Its presence has also been confirmed in the female reproductive tract of mice, where its occurrence was limited to the preimplantation period^73^. Next protein more abundant during REC-BL is KRT19 (keratin 19). Keratins are a family of structural proteins and are involved in epithelial cell development^74^.

After the start of egg laying, the protein composition of female fluids undergoes a further change, likely caused by egg production and the passage of eggs through the female reproductive tract. This is supported by the increased production of proteins that can be associated with the occurrence of an egg. Two proteins responsible for the variance between fluids are proteins from the serpin family—OVAL (ovalbumin) and OVALY (ovalbumin-related protein Y). Both proteins are also present in egg white which is predominantly composed of proteins that exhibit antimicrobial activity^75,76^, which was confirmed for OVAL^77,78^. There is also higher abundance of RBP (riboflavin-binding protein), whose precursor was already found in laying hens, where its occurrence was primarily associated with the transfer of vitamin B to the egg^79^. VTG1 (vitellogenin 1) is protein from vitellogenin family. Vitellogenin is the major precursor of egg yolk protein, and it has been documented in a wide range of oviparous species^80,81^. Vitellogenins have various roles including antioxidant activity^81,82^, so they could also prolong the viability of sperm. They are involved in various innate immune responses^81^ and they can protect the female from infections and bacteria. Furthermore, vitellogenin increases viscosity of the environment^83^. Higher viscosity of the female fluid can worsen the passage of sperm through the female reproductive tract, as it has a significant impact on sperm motility^84^. This phenomenon could ensure that only highly motile sperm reach the egg. FABP6 (fatty acid binding protein 6) was one of the most increased proteins in REC-L. Similar protein FABP5 was also abundant and significantly increased in mouse estrus^70^. The role of FABP6 in female reproduction is still not clear, but it is suggested to influence ovarian physiology. It is expressed in murine granulosa cells and is involved in ovulatory response to superstimulation^85^.

For searching candidate proteins of cryptic female choice, we looked for the differences common between NON and REC-BL, respectively REC-L. While the protein composition of the female reproductive tract changes after the start of laying, from the perspective of the cryptic female choice, it is still the environment in which sperm must to survive after copulation and transfer to the female reproductive tract, as passerine females continue to mate with males even during the egg-laying period^55–57^. Our analysis identified only three proteins that are significantly more abundant in both receptive phases (REC-BL, REC-L) compared to the two nonreceptive phases (PRE, POST). One of the significant proteins is RBP (riboflavin-binding protein). The main function of RBP is to transport riboflavin (vitamin B2). Riboflavin has two coenzyme forms—flavin mononucleotide (FNM) and flavin adenine dinucleotide (FAD), which play roles in fatty acid β-oxidation, the citric acid cycle, complex I and complex II of the electron transfer chains^86,87^. Therefore, riboflavin is an essential component of oxidative phosphorylation (OXPHOS), which provides energy for sperm^88^. Riboflavin also has antioxidant effects and can provide sperm with protection against reactive oxygen species as free radicals^89,90^, thereby extending the longevity of sperm. So, RBP could be involved in the selective uptake of sperm, as it is involved in the transport of riboflavin, which is an essential nutrient for sperm. The protein RARRES1 was mentioned above, and in the context of cryptic female choice, its protease inhibitor activity could be particularly important as well as the last protein, LOC100232544 (serine protease inhibitor 2.1 like). Protease inhibitors are essential components of sperm-egg interaction in mammals^50,91,92^, and they could play a similar role in other animal species. RARRES1 and LOC100232544 could be involved in the differential digestion of sperm, as they are both protease inhibitors. Protease inhibitors can protect proteins from being broken down, and they could be used by females to protect sperm from being digested by the female reproductive tract. These three proteins and their elevated levels in receptive female reproductive tracts suggest that they play a role in cryptic female choice, potentially influencing sperm survival and fertilization success.

We performed GO search using Gene Set Enrichment Analysis (GSEA) to determine the functions of proteins represented in the receptive phases (REC-BL and REC-L) of reproduction. We show that positively correlated GO terms (classified as activated) from the cloacal fluids of females in REC-BL phase are related to actin filament (proteins TPM1 and ACTA1). This could be associated with muscle function and therefore could be important for facilitating sperm passage into storage tubules or the uterus. In REC-L phase, activated GO term is lipid transporter activity (protein VTG1). Lipids are important compounds of sperm, providing structural stability and integrity, and could be essential for successful fertilization (reviewed in^93^). The suppression of the term sexual reproduction in REC-L is associated with three proteins—MYH9, PAFAH1B1 and TTR. However, these proteins may also be involved in other pathways, and their primary function may be completely different. In this study, proteins PAFAH1B1 and TTR are also involved in suppression of protein binding or signaling. Although the protein composition differs between the receptive phases and GO also changes, we observed some similarities between REC-BL and REC-L. We show that positively correlated GO terms associated with regulation of proteolysis are common to both phases of receptivity (proteins OVAL, OVALY, RARRES1, CST3, SERPINB10B in REC-BL; OVAL, OVALY, RARRES1 in REC-L). The regulation of proteolysis is essential for maintaining the proper functioning of the female reproductive system and is critical for processes such as ovulation and fertilization^94^. Moreover, protease inhibitors have been shown to possess potent antimicrobial activity by effectively suppressing the activity of bacterial proteases, thus preventing the proliferation of pathogenic microorganisms^95^, which could be of a special importance in defense against sexually transmitted diseases, especially in promiscuous species such as the barn swallow^96^.

This paper describes the temporal variation in the composition of female reproductive fluids in passerines. This has potentially significant implications for advancing our understanding of sperm-female reproductive tract interactions, cryptic female choice, and fertilization success of individual males. Our results demonstrate that the composition of cloacal fluids in passerines is not constant throughout the season, rather there are changes in the protein composition of the female reproductive tract during the receptive phase, akin to situations observed in mammals. These modifications probably serve to enhance the female readiness for reproduction (sperm reception and storage, fertilization, and oviposition). Our study has identified various proteins responsible for inducing these changes, and we have employed GO searching to elucidate the functional roles of the proteins expressed during the period of receptivity. Our proteomic analysis was able to find differences between the reproductive phases of female barn swallows based on the protein profile of the cloacal fluid. Contrary to expectations, the shifts in cloacal environment were due to small changes in relative abundance of many proteins, rather than a major changes in a few proteins. However, we were able to identify a few candidate proteins that may participate in female cryptic choice. These proteins could affect the survival and longevity of sperm and be involved in sperm-egg interactions. Our study represents an important first step in describing temporal dynamics in the female reproductive tract environment, deepening our understanding of the mechanisms behind cryptic female cryptic choice in internally fertilizing organisms.

### Supplementary Information

Supplementary Information.

## Data Availability

The mass spectrometry proteomics data have been deposited to the ProteomeXchange Consortium via the PRIDE^97^ partner repository with the dataset identifier PXD041798. The data supporting the conclusions of this article is also available as Supplementary Material.

## References

[1] Parker GA (1970). Sperm competition and its evolutionary consequences in the insects. Biol. Rev..

[2] Thornhill R (1983). Cryptic female choice and its implications in the Scorpionfly *Harpobittacus nigriceps*. Am. Nat..

[3] Firman RC, Gasparini C, Manier MK, Pizzari T (2017). Postmating female control: 20 years of cryptic female choice. Trends Ecol. Evol..

[4] Gasparini C, Pilastro A, Evans JP (2020). The role of female reproductive fluid in sperm competition. Philos. Trans. R. Soc. B Biol. Sci..

[5] Dean R, Nakagawa S, Pizzari T (2011). The risk and intensity of sperm ejection in female birds. Am. Nat..

[6] Lüpold S (2013). Female mediation of competitive fertilization success in *Drosophila melanogaster*. Proc. Natl. Acad. Sci..

[7] Fitzpatrick JL, Lüpold S (2014). Sexual selection and the evolution of sperm quality. Mol. Hum. Reprod..

[8] Birkhead TR, Møller AP (1993). Sexual selection and the temporal separation of reproductive events: Sperm storage data from reptiles, birds and mammals. Biol. J. Lin. Soc..

[9] Holt WV, Lloyd RE (2010). Sperm storage in the vertebrate female reproductive tract: How does it work so well?. Theriogenology.

[10] Bakst M, Wishart G, Brillard J-P (1994). Oviducal sperm selection, transport, and storage in poultry. Poult. Sci. Rev..

[11] Gasparini C, Pilastro A (2011). Cryptic female preference for genetically unrelated males is mediated by ovarian fluid in the guppy. Proc. R. Soc. B Biol. Sci..

[12] Pitnick, S., Wolfner, M. F. & Suarez, S. S. Ejaculate-female and sperm-female interactions. In *Sperm Biology: An Evolutionary Perspective* 247–304 (Academic Press, London, 2009). 10.1016/B978-0-12-372568-4.00007-0.

[13] Urbach D, Folstad I, Rudolfsen G (2005). Effects of ovarian fluid on sperm velocity in Arctic charr (*Salvelinus alpinus*). Behav. Ecol. Sociobiol..

[14] Villanueva-Diaz C, Vadillo-Ortega F, Kably-Ambe A, Diaz-Pérez MA, Krivitzky SK (1990). Evidence that human follicular fluid contains a chemoattractant for spermatozoa. Fertil. Steril..

[15] Oliveira RG, Tomasi L, Rovasio RA, Giojalas LC (1999). Increased velocity and induction of chemotactic response in mouse spermatozoa by follicular and oviductal fluids. Reproduction.

[16] Cramer ERA (2014). Testing a post-copulatory pre-zygotic reproductive barrier in a passerine species pair. Behav. Ecol. Sociobiol..

[17] Cramer ERA (2016). Sperm performance in conspecific and heterospecific female fluid. Ecol. Evol..

[18] Cramer ERA, Ålund M, McFarlane SE, Johnsen A, Qvarnström A (2016). Females discriminate against heterospecific sperm in a natural hybrid zone. Evolution.

[19] Poignet M (2022). Sperm morphology and performance in relation to postmating prezygotic isolation in two recently diverged passerine species. Sci. Rep..

[20] Ahammad MU (2013). Effects of fluid secreted from the uterus on duration of fertile egg production in hens, and survivability and penetrability of fowl sperm in vitro. J. Poult. Sci..

[21] Yeates SE (2013). Cryptic choice of conspecific sperm controlled by the impact of ovarian fluid on sperm swimming behavior. Evolution.

[22] Gasparini C, Evans JP (2013). Ovarian fluid mediates the temporal decline in sperm viability in a fish with sperm storage. PLoS ONE.

[23] Turner E, Montgomerie R (2002). Ovarian fluid enhances sperm movement in Arctic charr. J. Fish Biol..

[24] Gomendio M, Roldan ERS (2008). Implications of diversity in sperm size and function for sperm competition and fertility. Int. J. Dev. Biol..

[25] Knief U (2017). A sex-chromosome inversion causes strong overdominance for sperm traits that affect siring success. Nat. Ecol. Evol..

[26] Lahnsteiner F, Weismann T, Patzner R (1995). Composition of the ovarian fluid in 4 salmonid species: *Oncorhynchus mykiss*, *Salmo trutta *f* lacustris*, *Saivelinus alpinus* and *Hucho hucho*. Reprod. Nutr. Dev..

[27] Rosengrave P (2009). Chemical composition of seminal and ovarian fluids of chinook salmon (*Oncorhynchus tshawytscha*) and their effects on sperm motility traits. Comp. Biochem. Physiol. Part A Mol. Integr. Physiol..

[28] Ramm SA (2020). Seminal fluid and accessory male investment in sperm competition. Philos. Trans. R. Soc. B Biol. Sci..

[29] Zadmajid V, Myers JN, Sørensen SR, Ernest Butts IA (2019). Ovarian fluid and its impacts on spermatozoa performance in fish: A review. Theriogenology.

[30] Kholodnyy V, Gadêlha H, Cosson J, Boryshpolets S (2019). How do freshwater fish sperm find the egg? The physicochemical factors guiding the gamete encounters of externally fertilizing freshwater fish. Rev. Aquac..

[31] Swanson W, Vacquier V (2002). Reproductive protein evolution. Annu. Rev. Ecol. Evol. Syst..

[32] Lahnsteiner F (2000). Morphological, physiological and biochemical parameters characterizing the over-ripening of rainbow trout eggs. Fish Physiol. Biochem..

[33] Johnson SL (2020). Ovarian fluid proteome variation associates with sperm swimming speed in an externally fertilizing fish. J. Evol. Biol..

[34] Zhang H (2006). Use of proteomic analysis of endometriosis to identify different protein expression in patients with endometriosis versus normal controls. Fertil. Steril..

[35] Ma X (2007). Proteomic analysis of human ovaries from normal and polycystic ovarian syndrome. Mol. Hum. Reprod..

[36] Apichela SA (2015). Biochemical composition and protein profile of alpaca (*Vicugna pacos*) oviductal fluid. Anim. Reprod. Sci..

[37] Hatzirodos N (2019). Transcript abundance of stromal and thecal cell related genes during bovine ovarian development. PLoS ONE.

[38] Nakamura O (2020). Transport of maternal transthyretin to the fetus in the viviparous teleost *Neoditrema ransonnetii* (Perciformes, Embiotocidae). J. Comp. Physiol. B.

[39] Meng Y (2014). Effects of GnRH antagonist on endometrial protein profiles in the window of implantation. Proteomics.

[40] Muthukumar S (2014). Buffalo cervico-vaginal fluid proteomics with special reference to estrous cycle: Heat shock protein (Hsp)-70 appears to be an Estrus indicator1. Biol. Reprod..

[41] Černá M, Kuntová B, Talacko P, Stopková R, Stopka P (2017). Differential regulation of vaginal lipocalins (OBP, MUP) during the estrous cycle of the house mouse. Scientific Reports.

[42] Soleilhavoup C (2016). Proteomes of the female genital tract during the oestrous cycle. Mol. Cell. Proteomics.

[43] Griffith SC, Owens IPF, Thuman KA (2002). Extra pair paternity in birds: A review of interspecific variation and adaptive function. Mol. Ecol..

[44] Brouwer L, Griffith SC (2019). Extra-pair paternity in birds. Mol. Ecol..

[45] Birkhead TR, Brillard J-P (2007). Reproductive isolation in birds: postcopulatory prezygotic barriers. Trends Ecol. Evol..

[46] Froman D (2003). Deduction of a model for sperm storage in the oviduct of the domestic fowl (*Gallus domesticus*). Biol. Reprod..

[47] Bakst MR, Akuffo V (2007). Alkaline phosphatase reactivity in the vagina and uterovaginal junction sperm-storage tubules of turkeys in egg production: Implications for sperm storage. Br. Poult. Sci..

[48] Gautron J (2001). Ovotransferrin is a matrix protein of the hen eggshell membranes and basal calcified layer. Connect. Tissue Res..

[49] Riou C (2020). Avian uterine fluid proteome: Exosomes and biological processes potentially involved in sperm survival. Mol. Reprod. Dev..

[50] Riou C (2019). Proteomic analysis of uterine fluid of fertile and subfertile hens before and after insemination. Reproduction.

[51] Møller AP, Brohede J, Cuervo JJ, de Lope F, Primmer C (2003). Extrapair paternity in relation to sexual ornamentation, arrival date, and condition in a migratory bird. Behav. Ecol..

[52] Michálková R, Tomášek O, Adámková M, Kreisinger J, Albrecht T (2019). Extra-pair paternity patterns in European barn swallows *Hirundo rustica* are best explained by male and female age rather than male ornamentation. Behav. Ecol. Sociobiol..

[53] Møller AP, Mousseau TA, Rudolfsen G (2008). Females affect sperm swimming performance: A field experiment with barn swallows Hirundo rustica. Behav. Ecol..

[54] Møller AP (2009). Senescent sperm performance in old male birds. J. Evol. Biol..

[55] Møller AP (1985). Mixed reproductive strategy and mate guarding in a semi-colonial passerine, the swallow *Hirundo rustica*. Behav. Ecol. Sociobiol..

[56] Kempenaers B (1997). Does reproductive synchrony limit male opportunities or enhance female choice for extra-pair paternity?. Behavior.

[57] Mota PG, Hoi-Leitner M (2003). Intense extrapair behaviour in a semicolonial passerine does not result in extrapair fertilizations. Anim. Behav..

[58] Redfern CPF, Clark JA (2001). Ringers’ Manual.

[59] Redfern CPF (2008). Brood patches. Ringers’ Bull..

[60] Petrželková A (2015). Brood parasitism and quasi-parasitism in the European barn swallow *Hirundo rustica rustica*. Behav. Ecol. Sociobiol..

[61] Kuntová B, Stopková R, Stopka P (2018). Transcriptomic and proteomic profiling revealed high proportions of odorant binding and antimicrobial defense proteins in olfactory tissues of the house mouse. Front. Genet..

[62] Otčenášková T (2023). Comparative sperm proteomics in selected passerine birds reflects sperm morphology and mitochondrial metabolism. J. Vertebr. Biol..

[63] Cox J (2014). Accurate proteome-wide label-free quantification by delayed normalization and maximal peptide ratio extraction, termed MaxLFQ. Mol. Cell. Proteomics.

[64] Crawley MJ (2007). The R Book.

[65] Chawade A, Alexandersson E, Levander F (2014). Normalyzer: A tool for rapid evaluation of normalization methods for omics data sets. J. Proteome Res..

[66] Rohart F, Gautier B, Singh A, Cao K-AL (2017). mixOmics: An R package for ‘omics feature selection and multiple data integration. PLoS Comput. Biol..

[67] Pavelka N (2004). A power law global error model for the identification of differentially expressed genes in microarray data. BMC Bioinform..

[68] Wickham H (2016). Ggplot2: Elegant Graphics for Data Analysis.

[69] Wu T (2021). clusterProfiler 4.0: A universal enrichment tool for interpreting omics data. The Innovation.

[70] Matějková T, Dodoková A, Kreisinger J, Stopka P, Stopková R (2024). Microbial, proteomic, and metabolomic profiling of the estrous cycle in wild house mice. Microbiol. Spectr..

[71] Grande G (2015). Proteomic characterization of the qualitative and quantitative differences in cervical mucus composition during the menstrual cycle. Mol. Biosyst..

[72] Manstein DJ, Meiring JCM, Hardeman EC, Gunning PW (2020). Actin–tropomyosin distribution in non-muscle cells. J. Muscle Res. Cell Motil..

[73] Xiao S (2014). Differential gene expression profiling of mouse uterine luminal epithelium during periimplantation. Reprod. Sci..

[74] Karantza V (2011). Keratins in health and cancer: More than mere epithelial cell markers. Oncogene.

[75] Walczak J, Bocian S, Trziszka T, Buszewski B (2016). Hyphenated analytical methods in determination of biologically active compounds in hen’s eggs. Crit. Rev. Anal. Chem..

[76] Bílková B (2018). Domestic fowl breed variation in egg white protein expression: Application of proteomics and transcriptomics. J. Agric. Food Chem..

[77] Da Silva M (2015). The family secrets of avian egg-specific ovalbumin and its related proteins Y and X. Biol. Reprod..

[78] Sah N, Mishra B (2018). Regulation of egg formation in the oviduct of laying hen. World’s Poult. Sci. J..

[79] Bourin M (2012). Transcriptomic profiling of proteases and antiproteases in the liver of sexually mature hens in relation to vitellogenesis. BMC Genom..

[80] Hayward A, Takahashi T, Bendena WG, Tobe SS, Hui JHL (2010). Comparative genomic and phylogenetic analysis of vitellogenin and other large lipid transfer proteins in metazoans. FEBS Lett..

[81] Sun C, Zhang S (2015). Immune-relevant and antioxidant activities of vitellogenin and yolk proteins in fish. Nutrients.

[82] Lu C-L, Baker RC (1986). Characteristics of egg yolk phosvitin as an antioxidant for inhibiting metal-catalyzed phospholipid oxidations. Poult. Sci..

[83] Saunders DK, Fowler O, Smalley KN (2000). The effects of estradiol treatment on the blood viscosity of the bullfrog *Rana catesbeiana*. Trans. Kansas Acad. Sci..

[84] Schmoll T, Rudolfsen G, Schielzeth H, Kleven O (2020). Sperm velocity in a promiscuous bird across experimental media of different viscosities. Proc. R. Soc. B Biol. Sci..

[85] Duggavathi R (2015). The fatty acid binding protein 6 gene (Fabp6) is expressed in murine granulosa cells and is involved in ovulatory response to superstimulation. J. Reprod. Dev..

[86] Barile M, Giancaspero TA, Leone P, Galluccio M, Indiveri C (2016). Riboflavin transport and metabolism in humans. J. Inherit. Metab. Dis..

[87] Kuang W (2021). SLC22A14 is a mitochondrial riboflavin transporter required for sperm oxidative phosphorylation and male fertility. Cell Rep..

[88] Krisfalusi M, Miki K, Magyar PL, O’Brien DA (2006). Multiple glycolytic enzymes are tightly bound to the fibrous sheath of mouse spermatozoa. Biol. Reprod..

[89] Tang J (2014). Dietary riboflavin supplementation improve the growth performance and antioxidant status of starter white Pekin ducks fed a corn–soybean meal diets. Livest. Sci..

[90] Saedisomeolia, A. & Ashoori, M. Riboflavin in human health: A review of current evidences. in *Advances in Food and Nutrition Research* vol. 83, 57–81 (Elsevier, 2018). 10.1016/bs.afnr.2017.11.002 29477226

[91] Lee RK-K (2018). Expression of cystatin C in the female reproductive tract and its effect on human sperm capacitation. Reprod. Biol. Endocrinol..

[92] Li S-H (2018). Serine protease inhibitor SERPINE2 reversibly modulates murine sperm capacitation. Int. J. Mol. Sci..

[93] Surai PF (2001). Polyunsaturated fatty acids, lipid peroxidation and antioxidant protection in avian semen. Asian-Australas. J. Anim. Sci..

[94] Kiyozumi D, Ikawa M (2022). Proteolysis in reproduction: Lessons from gene-modified organism studies. Front. Endocrinol..

[95] Mine Y, Kovacs-Nolan J (2006). New insights in biologically active proteins and peptides derived from hen egg. World’s Poult. Sci. J..

[96] Poiani A, Wilks C (2000). Sexually transmitted diseases: A possible cost of promiscuity in birds?. The Auk.

[97] Perez-Riverol Y (2022). The PRIDE database resources in 2022: A hub for mass spectrometry-based proteomics evidences. Nucleic Acids Res..

