# Supplementary material for: Proteomic analysis reveals dynamic changes in cloacal fluid composition during the reproductive season in a sexually promiscuous passerine
Source: Sci Rep. 2024 Jun 20;14:14259. doi: 10.1038/s41598-024-62244-3 (PMC11190206; doi:10.1038/s41598-024-62244-3)

Supplementary information

for

**Proteomic analysis reveals dynamic changes in cloacal fluid composition during the reproductive season in a sexually promiscuous passerine**

Kristýna Míčková, Václav Jelínek, Oldřich Tomášek, Romana Stopková, Pavel Stopka & Tomáš Albrecht

**Supplementary Table 1.** Summary of Comp1, Comp2 and Comp3 loadings extracted from a Sparse Partial Least Squares Discriminant Analysis (sPLS-DA) of four phases (prereceptive – PRE, receptive before (REC-BL) and during (REC-L) egg laying, postreceptive – POST) in reproductive season. We used Comp2 and Comp3 for their higher informativeness (capture more differences between receptivity and nonreceptivity), even though Comp 1 explained more variability (26 %).

|  | **AUC** | **p value** |
| --- | --- | --- |
| **Comp1:** |  |  |
| PRE vs. others | 0.5644 | 0.540 |
| REC-BL vs. others | 0.6414 | 0.319 |
| REC-L vs. others | 0.8512 | 0.008 |
| POST vs. others | 0.7470 | 0.021 |
| **Comp2:** |  |  |
| PRE vs. others | 0.6515 | 0.149 |
| REC-BL vs. others | 0.6966 | 0.166 |
| REC-L vs. others | 1.0000 | <0.001 |
| POST vs. others | 0.8577 | <0.001 |
| **Comp3:** |  |  |
| PRE vs. others | 0.6136 | 0.280 |
| REC-BL vs. others | 1.0000 | <0.001 |
| REC-L vs. others | 1.0000 | <0.001 |
| POST vs. others | 0.8933 | <0.001 |

**Supplementary Table 2.** Summary of COMP1 and COMP2 loadings extracted from a Sparse Partial Least Squares Discriminant Analysis (sPLS-DA) of three phases (receptive before (REC-BL) and during (REC-L) egg laying, nonreceptive – NON) in reproductive season.

|  | **AUC** | **p value** |
| --- | --- | --- |
| **Comp1:** |  |  |
| NON vs. others | 0.8379 | 0.002 |
| REC-BL vs. others | 0.6897 | 0.181 |
| REC-L vs. others | 0.845 | 0.009 |
| **Comp2:** |  |  |
| NON vs. others | 1.000 | <0.001 |
| REC-BL vs. others | 0.7931 | 0.039 |
| REC-L vs. others | 0.9940 | <0.001 |

**Supplementary Figure 1.** Protein loadings from Sparse Partial Least Squares Discriminant Analysis (sPLS-DA) of four phases – prereceptive (PRE), receptive before (REC-BL) and during egg laying (REC-L) and postreceptive (POST). The proteins responsible for differences in protein composition of cloacal fluids among reproductive season are displayed using loadings from the sPLS-DA analysis. The proteins are ranked from bottom (highest importance) to top and the same colour code as in Fig. 1A was used.


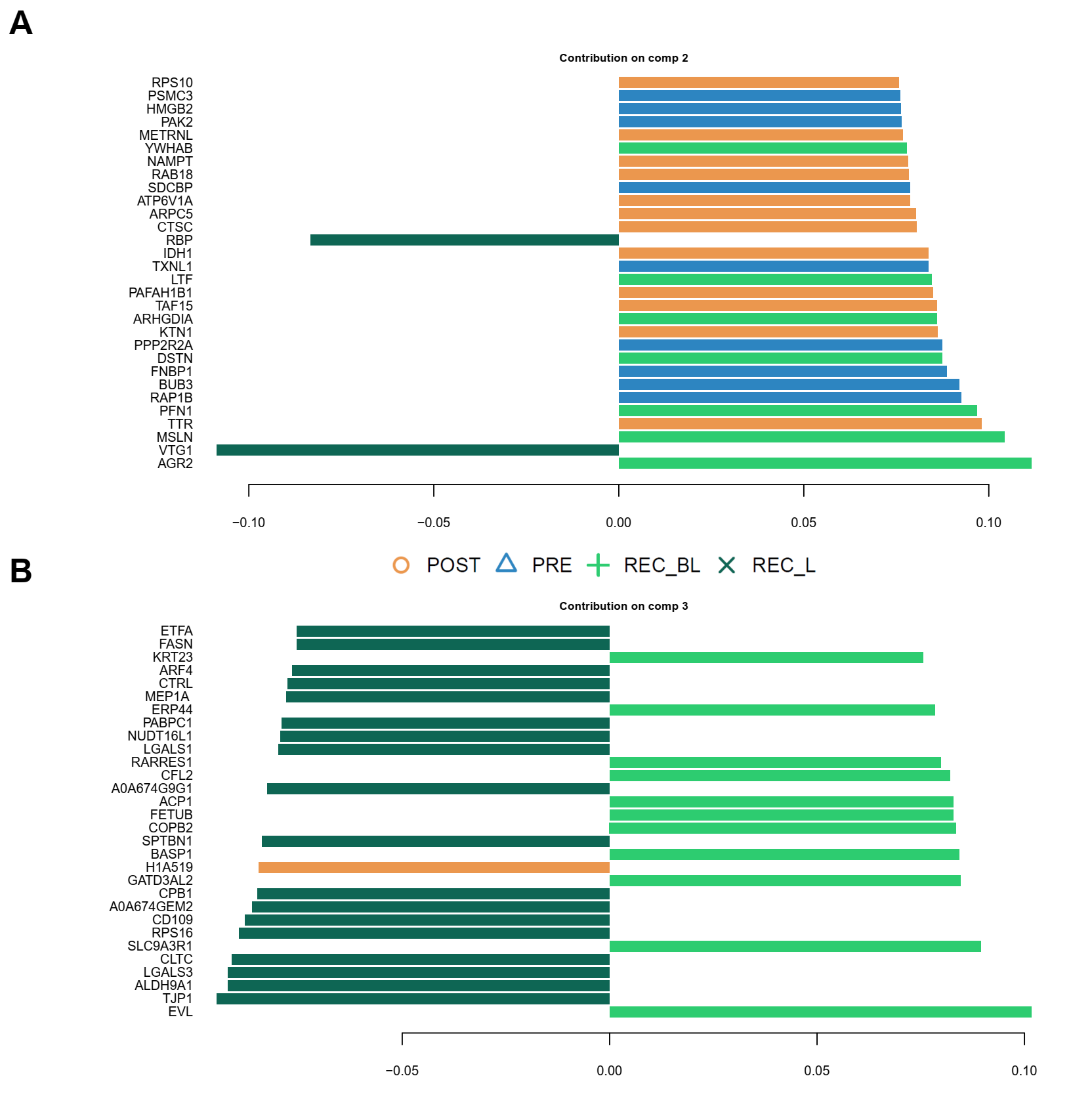


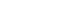

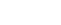


**Supplementary Figure 2.** Protein loadings from Sparse Partial Least Squares Discriminant Analysis (sPLS-DA) of three phases – nonreceptive (after merging PRE and POST in NON), receptive before (REC-BL) and during egg laying (REC-L). The proteins responsible for differences in protein composition of cloacal fluids among reproductive season are displayed using loadings from the sPLS-DA analysis. The proteins are ranked from bottom (highest importance) to top and the same colour code as in Fig. 1B was used.


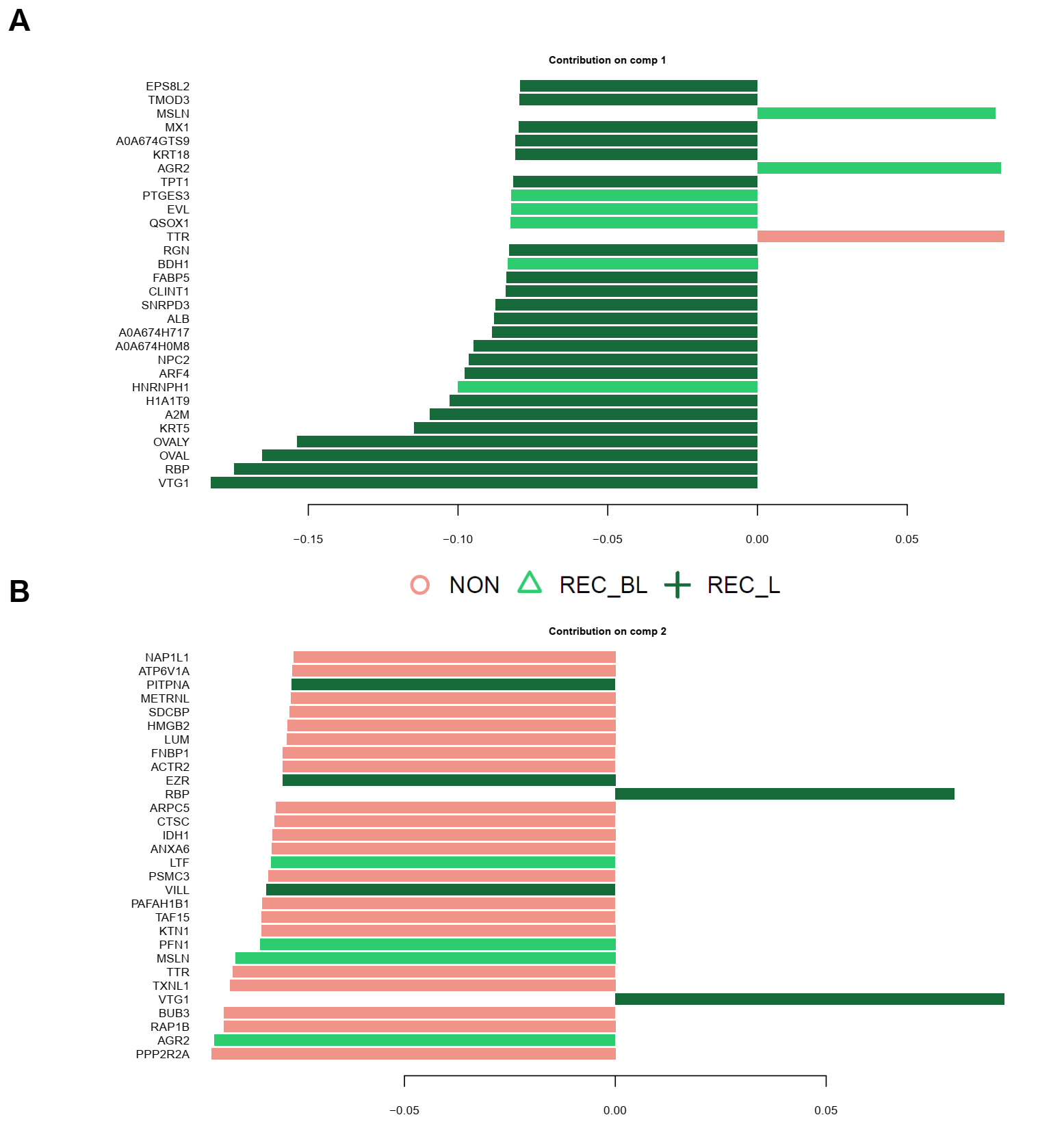


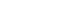

Supplement: Supplementary file 1 — Supplementary Information. [file 41598_2024_62244_MOESM1_ESM.docx]
